# Does the transfer of a poor quality embryo together with a good quality embryo affect the In Vitro Fertilization (IVF) outcome?

**DOI:** 10.1186/s13048-016-0297-9

**Published:** 2017-01-13

**Authors:** Eliana Muskin Wintner, Anat Hershko-Klement, Keren Tzadikevitch, Yehudith Ghetler, Ofer Gonen, Oren Wintner, Adrian Shulman, Amir Wiser

**Affiliations:** 1 IVF Unit, Department of Obstetrics and Gynecology, Meir Medical Center, Kfar Saba, Israel; 2 Affiliated with the Sackler Faculty of Medicine, Tel Aviv University, Tel Aviv, Israel

**Keywords:** Embryo transfer, Embryo implantation, Embryo quality, Live birth rate

## Abstract

**Background:**

IVF cycles which result in only one good quality embryo, and a second poor quality embryo present a dilemma when the decision involves transferring two embryos. The aim of this study was to evaluate whether a poor quality embryo has a negative effect on a good quality embryo when transferred along with a good quality embryo.

**Methods:**

We retrospectively evaluated in vitro fertilization (IVF) cycles involving single embryo transfers (SET) and double embryo transfers (DET). Embryo quality was divided into poor “P” and good “G” quality. The main outcome measures were: live birth, implantation rate, miscarriage rate, clinical pregnancy rate and multiple pregnancy ratio.

**Results:**

Six hundred three women were included. The study group consisted of 180 (29.9%) patients who had a double embryo transfer (DET) with one poor quality embryo and one good quality embryo (P + G). Control 1 group included 303 (50.2%) patients who had DET with two good quality embryos (G + G), and control 2 group consisted of 120 (19.9%) patients who had a single embryo transfer (SET) with one good quality embryo (G). Live birth rates were not significantly different when compared between study groups: 30.8% in the SET group (G), 27.2% in the (G + P) group and 33.7% in the (G + G) group. The SET group had the highest implantation rate (33.9%) compared to the DET groups (21.8% (G + P), 25.4% (G + G)) (*P* =0.022). The clinical pregnancy rate was 33.3% in the SET group (G), 33.3% in the (G + P) group, and 39.3% in the (G + G) group (*P* =0.39). The miscarriage rate was comparable in all groups.

**Conclusion:**

A poor quality embryo does not negatively affect a good quality embryo, when transferred together in a double embryo transfer.

**Electronic supplementary material:**

The online version of this article (doi:10.1186/s13048-016-0297-9) contains supplementary material, which is available to authorized users.

## Background

Embryo quality is one of the main predictors of success in IVF cycles [1, 2]. Many studies have shown a strong association between embryo morphology, implantation, and clinical pregnancy rates. In theory, the poor quality embryo has potential for a successful pregnancy. On the other hand, the poor quality embryo may lead to higher spontaneous abortions and overall decreased clinical pregnancies and live birth rates. A study done by Oron et al. [1] found that clinical pregnancy and live birth rates per transfer were nearly 2-fold higher with the transfer of a single good quality embryo than with the transfer of a poor quality embryo. However, once a clinical pregnancy was achieved, it had a similar chance of reaching live birth as a high quality embryo.

Additionally, it has been shown that double embryo transfers [DET] are associated with higher clinical pregnancy and live birth rates [3, 4], compared to single embryo transfers [SET]. Roberts et al. [3] reported that SETs have a one-third lower live birth rate then DETs in fresh cycles. Baruffi et al. [4] reported that fresh DET cycles have higher ongoing pregnancy and live births rates than SET.

Many IVF centers, therefore, increase the number of embryos transferred when the quality of embryos is decreased at cleavage stage. The potential situation of having only one good quality embryo with the second poor quality embryo presents a dilemma when the decision involves transferring two embryos. Should the poor quality embryo be transferred with the good quality embryo or does it have the potential to harm the implantation and pregnancy rate of the good embryo? A potential interaction exists between preimplantation embryos via paracrine factors, which affect the development and growth of the neighboring embryos [5]. In addition, it has been shown that the presence of poor quality embryos in the same culture decreases the overall blastulation rate of all embryos [6].

The aim of this study was to evaluate whether a poor quality embryo has a negative effect on a good quality embryo when transferred along with a good quality embryo.

## Methods

This is a retrospective cohort study encompassing all IVF and IVF ICSI cycles performed in a single university affiliated ART center between 2010-2015. Only cycles involving SET or DET of fresh non-donor oocytes were included. The study was approved by the local ethics committee.

### Embryo quality

Cleavage embryos were graded from one to four, based on percent fragmentation and cell counts: grade 4, equal-sized symmetrical cells with no fragmentation and 6-8 cells; grade 3, equal-sized symmetrical cells with less than 10% fragmentation and/or 4-5 cells; grade 2, non-symmetrical blastomers with 10-50% fragmentation, less than 4 cells; and grade 1 more than 50% fragmentation. Embryo quality grading was determined on the day of embryo transfer (day 3 or 5) : embryo quality was divided into poor quality “P” [grade 1 and 2] and good quality “G” [grade 3 and 4]. The study group consisted of patients who had DET with one “G” and one “P” embryos [group 1]. The control groups included; DET with two “G” embryos [control 1] and SET with one “G” embryo [control 2].

On Day 5, embryos were scored for blastocyst formation. Blastocysts were graded according to the size of the blastocyst, the assessment of the ICM and trophectoderm development [≥3BB] [7]. Good quality embryos [Grade 3-4] were defined as those where at least: [3] the blastocele completely fills the embryo (grade 3); the ICM is loosely grouped with several cells (grade B); and the trophoectoderm has very few cells forming a loose epithelium (grade B). Lower than 3BB quality embryos on Day 5 were defined as poor quality embryos [Grade 1 or 2]. The allocation to blastocyst versus cleavage transfer was based on the number and quality of cleavage embryos available on day 3. Blastocyst grading was performed by two trained embryologists, each with over 15 years of experience.

Pregnancy diagnosis; A quantitative pregnancy test [serum β-HCG] was taken 14 days after HCG administration. In case of a positive pregnancy test, a transvaginal ultrasound was performed 28-32 days after the embryo transfer and repeated as required. Clinical pregnancy was confirmed if a fetal heartbeat was observed by transvaginal ultrasound.

### Statistical analysis

Primary outcome measure was defined as live birth. Secondary outcome measures of the study were implantation rate, miscarriage rate, clinical pregnancy rate and multiple pregnancy ratio.

Analysis of data was performed using the SPSS 23.0 computer package [SPSS Inc., Chicago, IL]. Normally distributed data were analyzed by ANOVA. *χ* ^2^ or Fisher’s exact test were used for comparisons of rates and proportions. All P values were tested as two-sided and considered significant at less than 0.05.

A multivariate logistic regression model for clinical pregnancy was performed using the following variables: maternal age, paternal age, BMI, day 3 baseline FSH levels, E_2_ [estradiol] level on HCG day, embryo transfer day, cycle sequential number, and treatment group.

## Results

A total of 603 women were included in the study. 180 women had DET with one poor quality embryo and one good quality embryo [group 1(G + P)], 303 women had DET with two good quality embryos [control 1(G + G)], and 120 women had SET with one good quality embryo [control 2(G)]. The demographic characteristics are presented in Table 1. The patients in the SET group (control 2) were significantly younger and presented a significantly lower BMI compared to those of DET (group 1 and control 1; Table 1). Baseline FSH values showed a difference when compared across the groups (Table 1) though not clinically significant. The groups were comparable in gravity, parity, and etiology of infertility (Table 1). The cycle characteristics are presented in Table 2. E_2_ level on the day of hCG, number of oocytes retrieved, and fertilization rates were significantly higher in control 1 (G + G). The treatment outcome of all three groups is shown in Table 3. The implantation rates were significantly different between the three treatment groups; both control groups had a higher implantation rate compared to the study group. Despite this difference, the other main outcome measures (live birth, and clinical pregnancy rates, as well as the other outcome variables) were comparable between these groups. No difference in twinning rates was identified when comparing the two double transfer groups, while no cases of twins were registered in the SET group.

**Table 1:** Patients parameters presented by treatment group

	Group 1 (G + P) *N* = 180	Control 1 (G + G) *N* = 303	Control 2 (G) *N* = 120	*P* Value
Maternal Age (years)	35.1 ± 4.9	35.2 ± 4.7	32.2 ± 5.8	0.000
Maternal BMI (Kg/m^2^)	25.2 ± 5.3	25.6 ± 5.7	24.0 ± 4.4	0.035
Parity	0.6 ± 0.7	0.7 ± 0.8	0.6 ± 0.7	0.150
Maternal Smoking (%)	20.4	28.2	18.3	0.166
Baseline FSH (IU/L)	6.9 ± 2.8	7.2 ± 2.7	8.1 ± 3.5	0.002
Infertility (%)				0.174
- Primary	36	29.9	38.3	
- Secondary	64	70.1	61.7	
Etiology: (%)				0.171
- Ovulatory	5.6	6.7	0.8	
- Male Factor	43.5	41.7	41.2	
- Mechanical	12.4	14.7	18.5	
- Endometriosis	7.3	5.0	5.9	
- Uterine	2.8	1.0	0.0	
- Unexplained	28.2	31.0	33.6

**Table 2:** Cycle characteristics

	Group 1 (G + P) *N* = 180	Control 1 (G + G) *N* = 303	Control 2 (G) *N* = 120	*P* Value
Total Dose FSH (IU)	2601.6 ± 1360	2514.5 ± 1311	2333.0 ± 1385	0.233
E_2_ on HCG (Pg/ml)	1261.9 ± 650	1485.4 ± 758	1360.9 ± 788	**0.006**
Progesterone on HCG	0.6 ± 0.4	0.6 ± 0.3	0.7 ± 0.8	0.106
Endometrial Thickness (mm) on HCG	10.0 ± 2.6	10.4 ± 2.5	10.5 ± 2.7	0.155
Mode of Fertilization: patients (%)				0.442
- IVF	46(25.8)	100(33.3)	37(30.8)	
- ICSI	112(62.4)	168(56.0)	73(60.8)	
- Half ICSI	21(11.8)	32(10.7)	10(8.3)	
No. oocytes retrieved	7.5 ± 3.8	9.7 ± 5.3	7.8 ± 5.7	**0.000**
Fertilization Rate	51.8 ± 23.0	56.4 ± 20.4	51.3 ± 26.4	**0.034**

**Table 3:** Implantation rate and pregnancy outcomes

	Group 1 (G + P) *N* = 180	Control 1 (G + G) *N* = 303	Control 2 (G)w *N* = 120	*P* Value
Implantation Rate (%)	21.8	25.4	33.9	**0.022**
Clinical Pregnancy: patients (%)	60(33.3)	119(39.3)	40(33.3)	0.32
Twin pregnancy (out of cohort) : patients (%)^a^	17(9.5)	32(10.6)	0(0)	0.6
Live Birth Rate: patients (%)	49(27.2)	102(33.7)	37(30.8)	0.39
Other outcome variables: patients (%)				0.39
- Chemical	9(5.0)	22(7.3)	3(2.5)	
- Ectopic	3(1.7)	1(0.3)	2(1.7)	
- Miscarriage	11(6.1)	18(6.0)	8(6.7)	

^a^comparison performed only between group 1 and control 1

Due to the mentioned demographic and cycle dynamic differences between the groups, and in order to treat possible confounders, we designed a multivariate logistic regression model for live birth incorporating the following variables: maternal age, paternal age, BMI, day 3 baseline FSH levels, E2 level on HCG day, embryo transfer day [cleavage versus blastocyst], and treatment group (the number of picked ova was not included due to co-linearity with the maximal E2 levels). The treatment group was not found to be significantly related to the main outcome measure (OR 0.915, 95% CI 0.64-1.307, P value 0.625), neither was any of the other tested variables in the model (Additional file 1).

## Discussion

Our study was designed to question a common issue in the clinical practice of physicians dealing with IVF. What should we do with a poor quality embryo? If the decision is SET, than a good quality embryo has a higher chance to achieve pregnancy, though the poor quality embryo still has a chance for pregnancy [1]. If the decision is DET, and the option is combining a good quality embryo with a poor quality embryo, the concern is that the poor quality embryo can potentially harm the good one. Our cohort demonstrated, that the live birth rates were similar when a poor quality embryo was transferred along with a good quality embryo, when compared to both a single good quality embryo transferred on its own or to two good quality embryos transferred together. Therefore, the decision to transfer a poor quality embryo with a good quality embryo did not lower the chances for women to conceive and importantly, did not affect her chances for live birth. Conversely, it did not increase the chance either. The twin pregnancy rate of 9.5% in the study group shows that at least some of the embryos which were classified as poor were implanted and lead to a viable pregnancy. Interestingly, the implantation rate of the SET was higher compared to both DET groups. This may be due to the significantly younger age and lower BMI in the SET group vs the DET groups, yet the results did not change even after adjusting for these confounding factors.

Our results contradict a recent study performed by El-danasouri et al. [8], who found that transferring an impaired quality embryo along with a good quality embryo significantly lowered both the pregnancy and implantation rates, than transferring the good quality embryo alone.

Our findings did not change, even after adjusting for confounding factors such as maternal age, paternal age, BMI, day 3 baseline FSH levels, E_2_ level on HCG day and stage of the embryo. Additionally, miscarriage rates were similar in all groups. This is similar to other studies [1] which have shown that once a clinical pregnancy was achieved with poor quality embryo, it had a similar chance of reaching live birth as a high quality embryo. When choosing end-points for future studies, clinical pregnancy should be sufficient as we showed.

The crude live birth rate, and twin gestation rate were highest in the DET with two good quality embryos. Overall the twin live birth rate was 10.6% (G + G) vs 9.5% (G + P) vs 0% (G). Multiple pregnancies are high-risk pregnancies, and should be avoided if possible [9]. Based on our results, embryo transfers with one good and one poor quality embryo yielded higher multiple pregnancy rates than one good quality SET. In a case when a single embryo pregnancy is mandatory, the G + P option should also be avoided.

Our study results are somewhat limited by the way the embryos were graded. Despite technological advances, current morphological assessment of the embryo, even when performed by experienced embryologists, is subjective. This might partially explain our result regarding the pregnancy rate with poor quality embryos. Perhaps more advanced methods to evaluate embryos, such as time lapse and genetic screening or pre-gestational screening (PGS) [10, 11] will provide a better definition of good and poor quality embryos and help to better predict viable pregnancies.

Other limitations include the retrospective design. Ideally, a prospective randomized trial, which includes only patients who are candidates for DET and have only one good quality and one poor quality embryo, should be performed. However, this study would take a long time and would be very difficult to recruit patients who are undergoing DET to instead perform SET with only one good quality embryo. Given that, as far as we know, there is no clear data in the literature studied, we tried to reduce biases by creating two control groups.

According to our results, single embryo transfers (SET) should be the initial recommendation for patients. When good quality embryos are transferred, the implantation rate is higher and there is no difference in pregnancy rate. Double embryo transfers should be limited to patients with repeated implantation failure or repeated pregnancy loss. Yet, when two embryos are transferred, women can be reassured that the quality of the second embryo does not seem to affect the pregnancy rate or the risk of twin pregnancy.

## Conclusion

Based on our findings, a poor quality embryo does not negatively affect a good quality embryo, when transferred together in a double embryo transfer.

## Additional file

Additional file 1: Table S1. Regression model for live birth rate. (DOC 35 kb)

## Abbreviations

DET: Double embryo transfers
SET: Single embryo transfers
IVF: In vitro fertilization
“P”: Poor quality embryo
“G”: Good quality embryo
