# Supplementary material for: Does the transfer of a poor quality embryo together with a good quality embryo affect the In Vitro Fertilization (IVF) outcome?
Source: J Ovarian Res. 2017 Jan 13;10:2. doi: 10.1186/s13048-016-0297-9 (PMC5237322; doi:10.1186/s13048-016-0297-9)
Supplement: Additional file 1: Table S1. — Regression model for live birth rate. (DOC 35 kb) [file 13048_2016_297_MOESM1_ESM.doc]

Additional file 1: Table S1. regression model for live birth rate

| **variables in the Equation** | | | | | | | |
| --- | --- | --- | --- | --- | --- | --- | --- |
|  | | B | S.E. | Wald | df | Sig. | Exp(B) |
| Step 1^a^ | Maternalage | -.039 | .035 | 1.191 | 1 | .275 | .962 |
|  | PaternalAge | -.015 | .033 | .199 | 1 | .655 | .986 |
|  | BMI | -.003 | .026 | .015 | 1 | .902 | .997 |
|  | E2onHCG | .000 | .000 | .018 | 1 | .893 | 1.000 |
|  | HormonalProfileFSHUL | .027 | .055 | .245 | 1 | .621 | 1.027 |
|  | transferdaydichotom | .516 | .418 | 1.527 | 1 | .217 | 1.676 |
|  | Group | -.129 | .188 | .470 | 1 | .493 | .879 |
|  | Constant | .631 | 1.466 | .185 | 1 | .667 | 1.879 |
| a. Variable(s) entered on step 1: Maternalage, PaternalAge, BMI, E2onHCG, HormonalProfileFSHUL, transferdaydichotom, Group. | | | | | | | |
